# Supplementary figures and images for: Efficacy and safety of Atezolizumab plus Bevacizumab and Lenvatinib as first-line systemic therapies for hepatocellular carcinoma: A real-world study
Source: PLoS One. 2025 Dec 18;20(12):e0337351. doi: 10.1371/journal.pone.0337351 (PMC12714280; doi:10.1371/journal.pone.0337351)

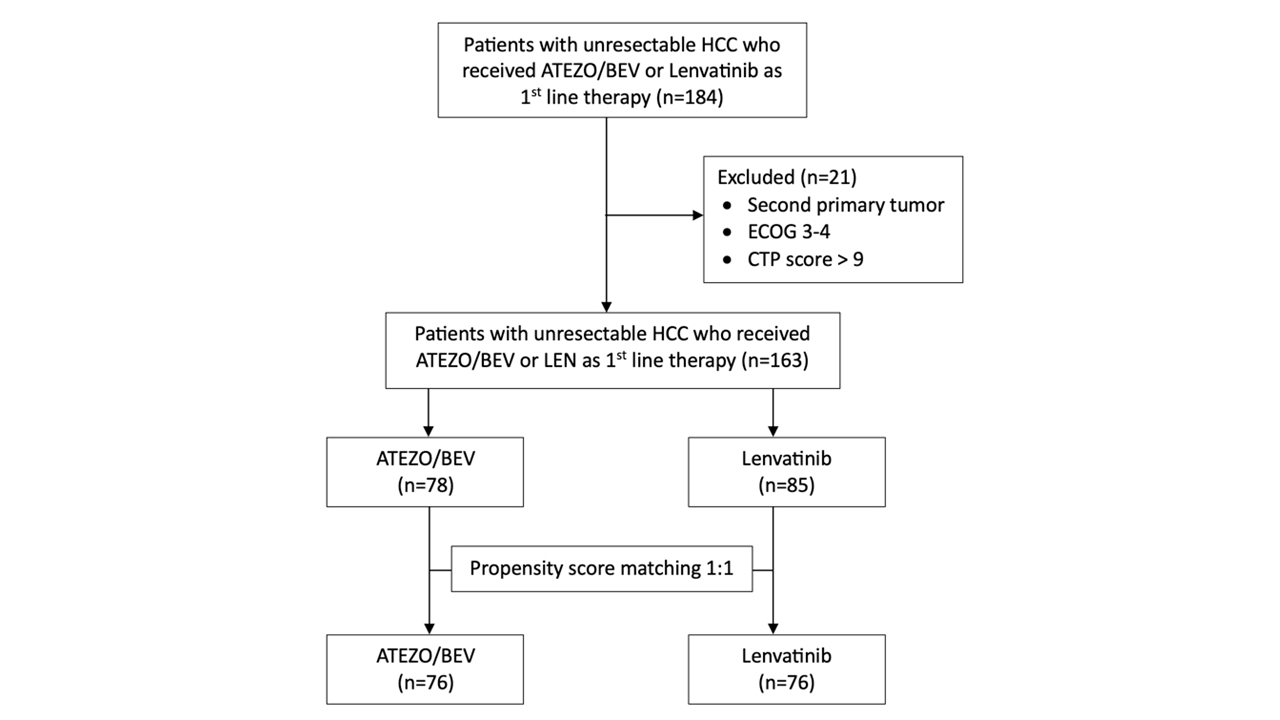

Supplement: S1 Fig — (TIF) [file pone.0337351.s001.tif]
