# Supplementary material for: Efficacy and safety of Atezolizumab plus Bevacizumab and Lenvatinib as first-line systemic therapies for hepatocellular carcinoma: A real-world study
Source: PLoS One. 2025 Dec 18;20(12):e0337351. doi: 10.1371/journal.pone.0337351 (PMC12714280; doi:10.1371/journal.pone.0337351)
Supplement: S1 Table — (DOCX) [file pone.0337351.s002.docx]

**S1 Table. Baseline characteristics after propensity score matching**

| **Clinical characteristic** | **Lenvatinib**  **(N=76)** | **ATEZO/BEV**  **(N=76)** | **P-value** |
| --- | --- | --- | --- |
| **Mean age, years** | 60.3 $\boldsymbol{\pm}$ 10.3 | 59.9$\boldsymbol{\pm}$ 13.2 | 0.93 |
| **Male** | 63 (82.9%) | 62 (81.6%) | 0.832 |
| **ECOG performance status**  ECOG 0  ECOG 1  ECOG 2 | 35 (46.1%)  33 (43.4%)  8 (10.5%) | 41 (53.9%)  34 (44.7%)  1 (1.3%) | 0.11 |
| **BW, kg** $\boldsymbol{\pm}$ **SD** | 64.8 $\boldsymbol{\pm}$ 12.1 | 64.9 $\boldsymbol{\pm}$ 12.4 | 0.75 |
| **T2DM** | 19 (25%) | 23 (30.3%) | 0.47 |
| **CKD** | 3 (3.9%) | 0 | 0.08 |
| **IHD** | 4 (5.3%) | 5 (6.6%) | 0.73 |
| **Child-Pugh Score**  Score <8  Score $\geq$8 | 63 (82.9%)  13 (17.1%) | 69 (90.8%)  7 (9.2%) | 0.05 |
| **Albumin, g/dL** | 3.5 $\boldsymbol{\pm}$ 0.6 | 3.5 $\boldsymbol{\pm}$ 0.5 | 0.57 |
| **Total bilirubin, mg/dL** | 1.5 $\boldsymbol{\pm}$ 1.2 | 1.4 $\boldsymbol{\pm}$ 1.0 | 0.93 |
| **AFP, ng/mL**  < 500  $\geq$ 500 | 53 (69.7%)  23 (30.3%) | 43 (58.9%)  30 (41.1%) | 0.17 |
| **Sodium, mEq/**$\boldsymbol{L}$ | 135.4 $\boldsymbol{\pm}$ 4.3 | 135.58 $\boldsymbol{\pm}$ 4.3 | 0.73 |
| **BCLC**  Stage B  Stage C | 25 (32.9%)  51 (67.1%) | 17 (22.4)  59 (77.6) | 0.15 |
| **Previous treatment** | 23 (30.3%) | 19 (25%) | 0.47 |
| **MWA/RFA** | 12 (15.8%) | 4 (5.3%) | 0.03* |
| **Resection** | 6 (7.9%) | 11 (14.5%) | 0.20 |
| **TACE** | 48 (63.2%) | 38 (50%) | 0.10 |
| **XRT** | 9 (11.8%) | 13 (17.1%) | 0.36 |
| **Y-90** | 2 (2.6%) | 9 (11.8%) | 0.03* |
| **Maximum tumor diameter, cm** | 7.7 $\boldsymbol{\pm}$ 5.7 | 8.2 $\boldsymbol{\pm}$6.0 | 0.65 |
| **Macrovascular invasion** | 30 (39.5%) | 31 (40.8%) | 0.51 |
| **Infiltrative lesion** | 14 (18.4%) | 13 (17.1%) | 0.83 |
| **Extrahepatic metastasis** | 30 (39.5%) | 39 (52%) | 0.12 |
| **Median follow-up time, months** | 5.5 (0.9-33.5) | 9.2 (0.7-36.9) | 0.02 |

Categorical variables were expressed as number and frequency, while continuous variable as mean ± SD or median (interquartile range)

Abbreviation: ATEZO/BEV; Atezolizumab plus bevacizumab, AFP; alpha-fetoprotein, ALBI; albumin-bilirubin score, BCLC; Barcelona Clinic liver Cancer, BW; body weight, CKD; chronic kidney disease, ECOG; Eastern Cooperative Oncology Group, IHD; ischemic heart disease, LEN; Lenvatinib, MWA/RFA; microwave ablation or radiofrequency ablation, PD; progressive disease, T2DM; type 2 diabetes mellitus, TACE; transarterial chemoembolization, XRT; radiation therapy, Y-90; Yttrium-90 radioembolization
