# Supplementary material for: Efficacy and safety of Atezolizumab plus Bevacizumab and Lenvatinib as first-line systemic therapies for hepatocellular carcinoma: A real-world study
Source: PLoS One. 2025 Dec 18;20(12):e0337351. doi: 10.1371/journal.pone.0337351 (PMC12714280; doi:10.1371/journal.pone.0337351)
Supplement: S2 Table — (DOCX) [file pone.0337351.s003.docx]

**S2 Table: The clinical response of patients during therapy after PSM**

|  | **ATEZO/BEV** | | **Lenvatinib** | | **P-value** |
| --- | --- | --- | --- | --- | --- |
|  | **N** | **%** | **N** | **%** |  |
| **At 12 weeks**  NE  PD  SD  PR  CR | 10  32  18  16  0 | 13.2  42.1  23.7  21.1  0 | 26  18  14  17  1 | 34.2  23.7  18.4  22.4  1.3 | 0.014* |
| **At 24 weeks**  NE  PD  SD  PR  CR | 0  6  12  12  0 | 0  20  40  40  0 | 0  10  5  8  0 | 0  43.5  21.7  34.8  0 | 0.148 |
| **At 36 weeks**  NE  PD  SD  PR  CR | 0  5  7  7  1 | 0  25  35  35  5 | 0  5  0  6  0 | 0  45.5  0  54.5  0 | 0.113 |
| **At 48 weeks**  NE  PD  SD  PR  CR | 0  4  2  4  3 | 0  30.8  15.4  30.8  23.1 | 0  2  0  4  0 | 0  33.3  0  66.7  0 | 0.311 |
| **ORR** | 18 | 23.7 | 15 | 19.7 | 0.555 |
| **DCR** | 12 | 15.8 | 29 | 38.2 | 0.867 |

Abbreviation: ATEZO/BEV; Atezolizumab plus bevacizumab, CR; complete response, DCR; disease control rate, NE; non-evaluable, ORR; objective response rate, PD; progressive disease, SD; stable disease
