# Supplementary material for: Efficacy and safety of Atezolizumab plus Bevacizumab and Lenvatinib as first-line systemic therapies for hepatocellular carcinoma: A real-world study
Source: PLoS One. 2025 Dec 18;20(12):e0337351. doi: 10.1371/journal.pone.0337351 (PMC12714280; doi:10.1371/journal.pone.0337351)
Supplement: S3 Table — (DOCX) [file pone.0337351.s004.docx]

**S3 Table: The standardized mean differences for baseline covariates before and after propensity-score matching**

|  | **Standardized Mean Differences** | |
| --- | --- | --- |
|  | **Before propensity-score matching** | **After propensity-score matching** |
| **Mean age, years** | 0.010 | 0.002 |
| **Male** | 0.020 | 0.030 |
| **ECOG performance status** | 0.604 | 0.013 |
| **BW, kg** | -0.007 | -0.0003 |
| **T2DM** | -0.154 | -0.053 |
| **CKD** | 0.269 | 0.039 |
| **IHD** | -0.082 | -0.013 |
| **Child-Pugh Score** | 0.470 | 0.053 |
| **Albumin, g/dL** | -0.134 | -0.092 |
| **Total bilirubin, mg/dL** | 0.035 | 0.0288 |
| **AFP ≥ 500 ng/mL** | -0.277 | -0.031 |
| **BCLC C** | -0.213 | -0.099 |
| **Previous treatment** | 0.993 | 0.053 |
| **Maximum tumor diameter, cm** | -0.023 | -0.015 |
| **Macrovascular invasion** | -0.066 | -0.028 |
| **Infiltrative lesion** | 0.016 | 0.034 |
| **Extrahepatic metastasis** | -0.217 | -0.052 |
