# Supplementary material for: Efficacy and safety of Atezolizumab plus Bevacizumab and Lenvatinib as first-line systemic therapies for hepatocellular carcinoma: A real-world study
Source: PLoS One. 2025 Dec 18;20(12):e0337351. doi: 10.1371/journal.pone.0337351 (PMC12714280; doi:10.1371/journal.pone.0337351)
Supplement: Attachment — Submitted filename: Point-by-point response to reviewers comments.docx [file pone.0337351.s006.docx]

**Point-by-point response to reviewer comments**

We sincerely appreciate all the valuable comments and suggestions which helped us improve the quality of the article. Below are the detailed, point-by-point responses to the reviewer’s comments. The manuscript has been updated for clarification and improvement

**Additional Editor Comments :**

**Dear Authors, the Reviewers have carefully analyzed your manuscript and concluded that there are some methodological issues that require re-evaluation in order to make this paper more scientifically sound and clear to the reader, as well as to improve reliability and reproducibility of the results achieved.**

**Particularly, more variable (like baseline laboratory markers or liver stiffness measurements, concurrent or previous treatments, comorbidities) should be included in the model, and more information about the patients who survived longer than the first 12 months from observation onset and about post-progression therapies should be included, so that differences in prognosis are more reliable and clinically useful.**

**Moreover, response evaluation and interpretation criteria should be reported with more details, considering that retrospective studies are more prone to interpretation biases than prospective ones.**

**Some figures and English text editing is also desirable.**

**Waiting for the revised version of your manuscript**

**We thank the reviewers and editor for their helpful comments and have revised the manuscript to address the points raised. The entire manuscript has undergone a thorough professional language edit to improve clarity, grammar, and style. Furthermore, all figures have been reviewed and enhanced to ensure high resolution and clear labelling for better readability.**

**Reviewer comments:**

**Reviewer #1: This study demonstrates that ATEZO/BEV significantly improves OS compared to LEN in patients with unresectable HCC, despite similar PFS, ORR, and DCR. Both treatments have comparable safety profiles. I have some advice for you.**

**1.Figures have disappeared at the bottom of this manuscripts.**

**2.Some grammatical errors in articles should be revised.**

**We thank the reviewer for the comments. We sincerely apologize for this mistake. This was a technical error that occurred during the final file compilation. We have carefully checked the revised manuscript and can confirm that all figures are now correctly placed and clearly legible at the end of the document. The entire manuscript has been thoroughly proofread and edited to correct all grammatical errors and improve sentence structure.**

**Reviewer #2: The authors present a retrospective real-world analysis comparing atezolizumab plus bevacizumab to lenvatinib as first-line systemic therapies in patients with unresectable hepatocellular carcinoma. The clinical relevance of this work is undeniable, as head-to-head randomized controlled trials directly addressing this therapeutic comparison are lacking, and clinicians often rely on retrospective evidence and propensity score-matched analyses to guide treatment selection. The manuscript is clearly written, and the study benefits from a relatively well-characterized cohort and careful application of propensity score matching. The findings, particularly the survival advantage with atezolizumab plus bevacizumab despite similar progression-free survival and objective response rates, are potentially meaningful. Nevertheless, there are several important methodological issues and interpretative limitations that must be addressed before the work can be considered for publication.**

**We thank the reviewer for the effort in evaluating our manuscript. We are grateful for the reviewer's positive assessment of our study's clinical relevance, the clarity of the manuscript, and the careful application of our propensity score matching methodology. It is encouraging that the reviewer recognizes the potential significance of our findings, especially given the current lack of head-to-head randomized controlled trials.**

**We also agree with the reviewer that there are important methodological issues and interpretative limitations that require careful consideration. Guided by the reviewer’s insightful feedback, we have carefully revised the manuscript to address each of these concerns.**

**1. The first major concern relates to the retrospective design and the inherent biases that arise despite the use of propensity score matching. The inclusion criteria, exclusion parameters, and data collection windows are not fully detailed. It is unclear whether all patients treated in the specified time frame were consecutively included, or whether certain subgroups might have been underrepresented. Without clear clarification, the risk of selection bias remains high.**

**Response**

**Thank you for your suggestions. We agree that the retrospective design inherently introduces potential biases, even with the use of propensity score matching. We have acknowledged this limitation in the discussion section of the manuscript. In addition, to provide greater clarity, we confirm that our study was designed to minimize this risk of selection bias by including all consecutive patients with unresectable hepatocellular carcinoma who initiated first-line systemic therapy with either Atezolizumab plus Bevacizumab or Lenvatinib at our institution between 2020 and 2023. The inclusion and exclusion criteria were applied systematically to the electronic medical records of every patient who started these treatments during the study period. This consecutive enrollment approach ensures that our study population is representative of the real-world patient cohort, thereby reducing the likelihood of underrepresenting specific subgroups. We have added a sentence to the Methods section to clarify this point.**

**We have revised the discussion section.**

**“First, the study had a retrospective design, which introduced inherent biases despite the application of PSM.” (Page 14, line 5-7)**

**We have revised the Method Section.**

**“This retrospective study consecutively included 163 patients with unresectable HCC who received first-line therapy with either LEN (n = 85) or ATEZO/BEV (n = 78) between 1 January 2020 and 31 December 2023 at King Chulalongkorn Memorial Hospital, Bangkok, Thailand. Eligible participants met the following criteria: 1) Adults aged 18 years or older with a confirmed diagnosis of unresectable HCC, based on typical imaging or histology (7), who received either LEN or ATEZO/BEV as first-line systemic therapy; 2) Child-Pugh class A or B liver function; and 3) An Eastern Cooperative Oncology Group (ECOG) performance status of 0 or 1. We excluded patients with: 1) a second primary cancer; 2) an ECOG performance status of 3–4; and 3) a CTP score > 9. Inclusion and exclusion criteria were systematically applied to all eligible patients identified through electronic medical records during the study period.” (Page 4, line 7-17)**

**2. Moreover, although the authors applied one-to-one nearest-neighbor matching, the choice of a caliper of 0.2 and the covariates included in the logistic regression model may not be sufficient to account for important clinical confounders. For example, baseline laboratory markers such as platelet count, neutrophil-to-lymphocyte ratio, or liver stiffness measurements, which are increasingly recognized as important prognostic determinants in hepatocellular carcinoma, were not considered. Similarly, the presence of comorbidities, previous local therapies, or concomitant supportive care measures could have significantly impacted survival outcomes. A sensitivity analysis including additional variables, or at minimum a discussion of why these parameters were excluded, would strengthen the validity of the comparisons.**

**Response**

**We would like to thank you for your helpful suggestions. We agree that including additional covariates strengthens the model and helps to account for potential clinical confounders. In response to this feedback. We have added comorbidities (diabetes mellitus, ischemic heart disease and chronic kidney disease), a history of previous locoregional therapies, platelet count and NLR in the logistic regression analysis (Revised Table 4).**

**We acknowledge that liver stiffness is a valuable prognostic indicator. However, due to the retrospective nature of our study, these data were not uniformly documented for the entire cohort, which precluded their inclusion in the PSM analysis without introducing significant amounts of missing data. Furthermore, the liver stiffness values as evaluated by transient elastography can be confounded by the tumor burden, especially in patients with large or infiltrative HCC. We have mentioned these points in the limitations of the study.**

**We have revised Table 4.**

**We have revised the results section.**

**“The most common comorbidities included type 2 diabetes mellitus in 44 patients (27%), ischemic heart disease in 9 patients (5.5%), and chronic kidney disease in 3 patients (1.8%).” (Page 6, line 5-7)**

**“Regarding prior therapies, transarterial chemoembolization (TACE) was the most common, having been performed in 91 patients (55.8%). Other previous treatments included microwave or radiofrequency ablation (MWA/RFA) (11%, n=18), surgical resection (11%, n=18), external radiation therapy (XRT) (14.7%, n=24), and Yttrium-90 (Y-90) radioembolization (6.7%, n=11).” (Page 6, line 14-18)**

**We have revised the discussion section.**

**“Third, while our PSM analysis adjusted for several key clinical variables, we acknowledge the potential for residual confounding from unmeasured factors. Baseline liver stiffness measurement was not included in the model due to inconsistent data availability. Moreover, the reliability of liver stiffness measured by transient elastography can be compromised in this patient population, as the values may be confounded by the tumor, especially in patients with a high tumor burden or infiltrative lesions.” (Page 14, line 11-16)**

**3. Another methodological limitation is the relatively short follow-up period, with a median under twelve months. The observed median overall survival in both arms is substantially lower than in pivotal clinical trials, which may reflect the advanced stage and higher tumor burden of the enrolled population, but may also stem from limited observation time and immature survival data. This discrepancy raises concerns about the robustness of the survival estimates. The authors should provide more granular details on censoring, the proportion of patients alive at last follow-up, and whether survival curves plateaued or continued to decline steeply beyond one year. Without this information, the survival advantage of atezolizumab plus bevacizumab over lenvatinib might be overestimated.**

**Response**

**We thank the reviewer for raising this important point regarding the follow-up period and the observed survival outcomes. We agree that the relatively short follow-up period is a key limitation of our study and warrants a more detailed discussion. The discrepancy in median overall survival compared to the pivotal IMbrave 150 and REFLECT trials may be explained by a higher tumor burden in this study or limited observation time and immature survival data. We also acknowledge in our limitations that longer follow-up is necessary to obtain mature survival data.**

**We have revised the methodology section for more clarification. Overall survival was defined as the time from the date of treatment initiation to the date of death from any cause, whereas progression-free survival was defined as the time from treatment initiation to the date of documented disease progression or death, whichever occurred first. The data cut-off for this analysis was June 30, 2024. For the overall survival analysis, data for patients who were alive at the data cut-off date were censored at that date. Patients lost to follow-up before this date were censored at their last known date of contact. The proportion of patients alive in each treatment arm at the time of the final data cut-off was calculated to assess data maturity.**

**We have revised the method section.**

“Overall survival (OS) was defined as the time from the date of treatment initiation to the date of death from any cause, whereas progression-free survival (PFS) was defined as the time from treatment initiation to the date of documented disease progression or death, whichever occurred first. The data cut-off for this analysis was June 30, 2024. For the OS analysis, data for patients who were alive at the data cut-off date were censored at that date. Patients lost to follow-up before this date were censored at their last known date of contact. The proportion of patients alive in each treatment arm at the time of the final data cut-off was calculated to assess data maturity.” (Page 5, line 16-23)

**We have revised the discussion section.**

“The shorter OS and PFS observed in our study compared to IMBRAVE 150 and REFLECT study, might be attributable to the more advanced baseline liver disease and tumor burden. Furthermore, the relatively short follow-up period might resulted in immature survival data, as evidenced by the Kaplan-Meier curves for OS, which decline steeply without plateauing. This highlights the need for longer-term follow-up to confirm the durability of the observed survival benefit.” (Page5, line 6-11)

**4. Treatment exposure and post-progression therapies also represent an area requiring greater detail. The discussion acknowledges that socioeconomic barriers influenced second-line treatment access, yet the manuscript does not provide clear data on how many patients in each arm received subsequent systemic therapy, locoregional salvage interventions, or best supportive care only. Given the well-documented impact of treatment sequencing on overall survival in hepatocellular carcinoma, the omission of these details limits interpretability. A breakdown of post-progression therapies, stratified by treatment arm, would allow readers to better understand whether the survival difference observed is attributable to first-line efficacy or subsequent management differences driven by cost and accessibility.**

**Response**

We thank the reviewer for this important point. We agree that post-progression therapies have a significant impact on overall survival and that a lack of these data can limit the interpretability of our findings. We have reviewed our electronic patient records to collect this information and have included it in the results section.

**We have revised the method section**

“Analysis of post-progression management revealed significant differences between the treatment arms. Patients who progressed on ATEZO/BEV were significantly more likely to receive subsequent systemic therapy compared to those on LEN (35.7% vs. 18.2%; P = 0.04). Conversely, a significantly higher proportion of patients in the Lenvatinib group received only best supportive care (52.2% vs. 30.9%; P = 0.01). The use of locoregional salvage interventions was comparable between ATE/BEV and LEN groups (33.3% vs. 29.5%, p=0.57).” (Page 9, line 10-15)

**We have revised the discussion section**

“A significant disparity was observed in the receipt of second-line systemic therapy: 35.7% of patients who progressed on ATE/BEV received subsequent treatment, compared to only 18.2% of those in the LEN group (P = 0.04). This difference likely reflects financial barriers that limited access to more expensive second-line options for patients who had failed first-line LEN.” (Page 13, line 22-26)

**5.The methods for assessing response also warrant scrutiny. The manuscript reports that mRECIST criteria were applied, but it is not specified whether evaluations were conducted by blinded radiologists, whether inter-observer variability was assessed, or whether central review was performed. These details are not trivial, since retrospective real-world imaging assessments can be inconsistent, and progression-free survival may be particularly sensitive to variations in interpretation. Additionally, while objective response rate and disease control rate were reported, duration of response—an endpoint of growing importance in immunotherapy-based regimens—was not addressed. Including this analysis would have enhanced the understanding of how responses differ qualitatively between the two regimens.**

**Response**

**Thank you for your suggestions. We agree that the methods for response assessment should be described in greater detail. The radiological evaluations were performed by two independent radiologists blinded to clinical outcomes, with a third radiologist adjudicating any discrepancies. We also agree that analyzing the Duration of Response (DOR) is essential for understanding the quality and durability of responses, particularly with immunotherapy. However, we found that the number of patients who achieved an objective response (complete or partial response) in our cohort was too small to allow for a statistically meaningful calculation of median DoR. Therefore, while we could not perform the formal analysis, we have acknowledged this as a key limitation of our study.**

**We have revised the method section.**

**“The radiological evaluations were performed by two independent radiologists blinded to clinical outcomes, with a third radiologist adjudicating any discrepancies.” (Page 5, line 11-13)**

**We have revised the discussion section.**

**“Fourth, a formal analysis of the duration of response was precluded by the limited number of patients who achieved a complete or partial response.” (Page 14, line 16-18)**

**6. The handling of alpha-fetoprotein and other tumor biomarkers deserves more thorough exploration. The multivariate analysis confirms alpha-fetoprotein ≥500 ng/mL and tumor size as independent predictors of poor overall survival, which is consistent with prior literature. However, (DCP), another validated biomarker with both diagnostic and prognostic implications, was not included in the dataset. Given its widespread use in Asian populations, the omission is striking and should be at least acknowledged. Furthermore, alpha-fetoprotein dynamics during treatment, rather than baseline levels alone, could provide valuable insight into biological activity and treatment efficacy. A sensitivity analysis considering AFP response at eight to twelve weeks might have clarified whether biochemical response aligned with radiologic and survival outcomes.**

**Response**

Thank you for your suggestion. We agree that DCP and other biomarkers could provide more valuable insights. However, DCP levels were not routinely measured in our center, as this test is not covered under the Thai healthcare reimbursement policy. We have mentioned this point in the discussion section.

**We have revised the discussion section.**

“Finally, our study did not incorporate an analysis of biological or immune-based biomarkers to stratify patient outcomes. The future of HCC management undoubtedly lies in biomarker-driven treatment algorithms that can predict which patients are most likely to benefit from specific systemic therapies (Shahini E. Int J Mol Sci. 2023). The retrospective design of our study precluded the systematic collection of tissue for assessing the tumor immune microenvironment, and key circulating biomarkers like des-gamma-carboxy prothrombin were not consistently available. Future prospective, real-world studies are urgently needed to integrate these multi-faceted biomarker analyses with clinical data to create more precise, individualized treatment strategies.” (Page 14, line 18-26)

**7. The study presents safety data, but the adverse event reporting is somewhat superficial. It is not indicated whether adverse events were prospectively recorded in electronic health records or retrospectively extracted, and the grading system, while referenced, is not explicitly described in the results. Importantly, the lack of reporting on immune-related adverse events such as hepatitis, colitis, or pneumonitis, even if absent, is problematic when discussing immunotherapy-based combinations. Moreover, adverse events leading to dose reduction or permanent discontinuation should be highlighted, since tolerability and treatment persistence critically affect outcomes in real-world practice.**

**Response**

We thank the reviewer for the feedback on our safety data reporting. We have substantially revised the manuscript to address all the points raised. The adverse events were retrospectively extracted from electronic health records. The data on immune-related events, dose reduction and permanent discontinuation were reviewed and added into the revised manuscript.

**We have revised the method section.**

“The AEs were retrospectively extracted from electronic health records.” (Page 5, line 6-7)

**We have added details on immune-related events, dose reduction and permanent discontinuation in Table 3.**

**We have revised the result section.**

“The rate of treatment-related adverse events causing discontinuation was similar in both groups. While treatment-related adverse events led to dose reduction in 7.9% of patients on Lenvatinib, no patients on ATEZO/BEV required a dose reduction (P=0.027).” (Page 9, line 21-24)

**We have revised the discussion section.**

“Regarding AEs, there were no significant differences in adverse events and overall rates of treatment discontinuation between LEN and ATE/BEV groups, their AE profiles varied significantly. Patients receiving LEN treatment had a significantly higher incidence of decreased appetite (21.1% vs. 6.6%; P = 0.010), diarrhea (13.2% vs. 0%; P = 0.001), and fatigue (32.9% vs. 10.5%; P = 0.001). Furthermore, treatment-related AEs resulted in significantly more dose reductions for the LEN group compared to the ATEZO/BEV groups (7.9% vs 0%, p = 0.027). ” (Page 13, line 28-34)

**8. From a statistical standpoint, the use of propensity score matching is appropriate, but the presentation of results would benefit from additional clarity. Balance diagnostics, such as standardized mean differences for baseline covariates before and after matching, are not reported. Without this, it is difficult to assess whether the matching adequately eliminated systematic imbalances.**

**Response**

Thank you for your suggestion. We agree that reporting balance diagnostics is essential for demonstrating the success of the propensity score matching procedure. We have performed this analysis and included the results in a new supplement table 3 (S3 Table). The standardized mean differences (SMD) for all baseline covariates were substantially reduced after matching. The absolute SMD for nearly all covariates was well below the recommended threshold of 0.1, indicating that a good balance was achieved between both groups and that systematic imbalances were adequately eliminated.

**We have revised the result section and added the S3 Table.**

**“**The standardized mean differences (SMD) for baseline covariates before and after matching were shown in the S3 Table. The SMD for all baseline covariates was substantially reduced after matching. The absolute SMD for nearly all covariates was well below the recommended threshold of 0.1, indicating that a good balance was achieved between both groups and that systematic imbalances were adequately eliminated.” (Page 9, line 5-9)

**9. Furthermore, the authors rely heavily on p-values for interpreting differences, yet confidence intervals are often wide and overlap substantially, particularly for progression-free survival. The discussion should place greater emphasis on the uncertainty of these estimates rather than drawing strong conclusions based on borderline statistical significance.**

**Response**

We sincerely thank the reviewer for their careful attention to detail. Their comment prompted us to re-examine our analysis, where we identified a typographical error. In the original manuscript, the hazard ratio for overall survival before propensity score matching was incorrectly stated, having been erroneously duplicated from the progression-free survival analysis. We have now corrected the sentence to report the accurate OS hazard ratio. The conclusion that median PFS did not differ significantly between the groups remains unchanged. We apologize for this mistake and appreciate the opportunity to correct the manuscript.

**We have revised the result section.**

“In contrast, median OS was significantly longer in the ATEZO/BEV group than in the LEN group (12.25 months vs. 7.49 months; p = 0.036, HR = 0.66, 95%CI: 0.45-0.97)” (Page 6, line 28-30)

**9. A broader issue is the limited generalizability of the findings. The cohort is derived from a single tertiary hospital in Thailand, with a very high prevalence of viral hepatitis as the underlying etiology. While this is representative of regional epidemiology, hepatocellular carcinoma in Western populations is increasingly driven by metabolic dysfunction-associated steatotic liver disease, which may respond differently to immunotherapy or anti-angiogenic agents. The authors should more clearly acknowledge that extrapolation of their findings to non-viral HCC should be cautious.**

**Response**

We would like to thank you for the comment on the generalizability. We agree that our study cohort, being from a single tertiary hospital in Thailand, has a high prevalence of viral hepatitis - associated hepatocellular carcinoma (79.4%). As the reviewer correctly points out, this reflects the regional epidemiology but differs from Western populations, where metabolic dysfunction-associated steatotic liver disease is an increasingly common driver of HCC.

We also noted that the current evidence supporting different treatment efficacy based on etiology is still emerging. For instance, a recent meta-analysis of six large randomized controlled trials reported a significant survival advantage for modern systemic therapies across both non-viral and viral etiologies (*Meyer T.* J Hepatol 2023). Nevertheless, to ensure this important context is clear to the reader, we have expanded upon this point in the limitations section of our manuscript.

**We have revised the discussion section.**

“Second, the generalizability of our findings is limited by the single-center design. The study cohort is from Thailand and has a high prevalence of viral hepatitis as the underlying etiology for HCC (79.4%). Given that this differs significantly from Western populations, where non-viral HCC is more common, caution is advised when extrapolating our results to patients with different underlying liver diseases.” (Page 14, line 6-11)

**10. Finally, the study leaves unaddressed the pressing need for integrated biomarker-driven algorithms in hepatocellular carcinoma. Real-world comparative studies such as this one are valuable, but they risk remaining descriptive unless coupled with efforts to stratify patients more precisely based on molecular or immune signatures. The immunomicroenvironment of hepatocellular carcinoma is highly heterogeneous, with some tumors characterized by immune exclusion and others by immune infiltration. Atezolizumab plus bevacizumab might preferentially benefit the latter group through synergistic enhancement of anti-tumor immunity and vascular normalization. Incorporating assessments of immune infiltrates, PD-L1 expression, or circulating immune markers would have allowed the authors to begin addressing which patients derive the greatest benefit. Such analyses could ultimately feed into decision-support algorithms that combine clinical features, biomarkers such as AFP and DCP, and immunomicroenvironmental characteristics to guide individualized first-line therapy selection (please refer to PMID: 36901717 and expand).**

**Response**

We are very grateful to the reviewer for this comment. We agree that the development of integrated, biomarker-driven algorithms is a critical and pressing need for personalizing the management of hepatocellular carcinoma. The reviewer correctly points out that our study would have been greatly strengthened by stratifying patients based on molecular or immune signatures. Unfortunately, the retrospective nature of our study presented significant barriers to performing such analyses. The systematic collection of tissue samples required for assessing the tumor immune microenvironment was not feasible for this cohort. Furthermore, as the reviewer suggests, incorporating biomarkers such as AFP and DCP is vital. While we did analyze AFP, we were unable to include DCP. As noted in our manuscript, DCP levels were not routinely measured at our center because the test is not covered by the Thai healthcare reimbursement policy. We have added a new paragraph to the limitations section of our manuscript to highlight the absence of biomarker analysis and to underscore the importance of this area for future research.

**We have revised the discussion section.**

“Finally, our study did not incorporate an analysis of biological or immune-based biomarkers to stratify patient outcomes. The future of HCC management undoubtedly lies in biomarker-driven treatment algorithms that can predict which patients are most likely to benefit from specific systemic therapies (*Shahini E*. Int J Mol Sci. 2023). The retrospective design of our study precluded the systematic collection of tissue for assessing the tumor immune microenvironment, and key circulating biomarkers like des-gamma-carboxy prothrombin were not consistently available. Future prospective, real-world studies are urgently needed to integrate these multi-faceted biomarker analyses with clinical data to create more precise, individualized treatment strategies.” (Page 14, line 18-26)

**11. In conclusion, this study makes a timely contribution to the literature by comparing atezolizumab plus bevacizumab with lenvatinib in unresectable hepatocellular carcinoma, and its findings align with the growing perception of immunotherapy-based combinations as superior in terms of survival benefit. However, methodological shortcomings, limited biomarker integration, and the absence of granular data on follow-up and subsequent therapies restrict the strength of the conclusions. Addressing these issues would substantially enhance the manuscript’s impact. Looking forward, the integration of tumor biology, circulating biomarkers, and immunomicroenvironment profiling into real-world datasets will be critical to developing robust algorithms that can guide screening, surveillance, and first-line treatment strategies in hepatocellular carcinoma.**

**Response**

We sincerely thank the reviewer for their thoughtful and comprehensive summary of our work. We are encouraged that they found the study to be a timely contribution and appreciate their insightful critique. We agree with their assessment of the study's limitations and, in response, have expanded the revised manuscript to address these points more explicitly. Finally, we concur entirely with the reviewer's forward-looking perspective. The integration of tumor biology and circulating biomarkers is crucial for advancing HCC treatment. We have added a sentence to our conclusion to reflect this, underscoring that future research must focus on these areas to develop the robust algorithms needed to guide personalized therapy.

We are confident that these revisions, guided by the reviewer's constructive feedback, have substantially strengthened the manuscript's impact and clarity. We appreciate the opportunity to improve our work.
